# ACCEPTABILITY OF A REMINISCENCE THERAPY TABLET APP: IMPACT ON RESIDENTIAL CARE STAFF OF PERSONS LIVING WITH DEMENTIA

**DOI:** 10.1093/geroni/igad104.2172

**Published:** 2023-12-21

**Authors:** Silvia Orsulic-Jeras, Ashlee Cordell, Sara Powers, Farida Ejaz, Lisbeth Sanders

**Affiliations:** Benjamin Rose Institute on Aging, Cleveland, Ohio, United States; Benjamin Rose Institute on Aging, Cleveland, Ohio, United States; Benjamin Rose Institute on Aging, Cleveland, Ohio, United States; Benjamin Rose Institute on Aging, Cleveland, Ohio, United States; LifeBio, Marysville, Ohio, United States

## Abstract

With dementia prevalence rapidly on the rise and no viable pharmacological treatments currently available, the development of accessible, efficacious, and low-cost non-pharmacologic interventions for persons living with dementia (PLWD) has become critical. Reminiscence therapy (RT) and life story can be built into residential care provision to provide person-centered solutions that elicit conversation, engagement, and socialization with staff. RT tools can also help inform care staff about residents’ lives and preferences to provide higher quality and meaningful interactions. This paper details the development and evaluation of LifeBio Memory, a tablet application designed to record the life stories and care preferences of PLWD in residential care communities. Care staff (n = 60) from 10 communities in Ohio, including memory care, assisted living and nursing homes, were trained to implement LifeBio Memory in their organizations. A pre-post design was used with staff, mainly comprised of life enrichment, administration, social work, rehab, and direct care staff. Outcomes presented include but will not be limited to: 1) acceptability, feasibility, and satisfaction with the tablet app, 2) staff knowledge of resident care preferences and interests, 3) utilization of person-centered care practices, 4) quality of communication between the resident and themselves and other staff, 5) their current understanding of that resident’s life history, and 6) level of tension and frustration with the resident. Discussion will focus on the need for sustainable RT interventions that promote person-centered care for PLWD and ways in which technological advancements can be integrated into residential care staff workflow.

